# The Prognostic Significance of the Second Predominant Histological Pattern in Resected Early-Stage Lung Adenocarcinoma: A Retrospective Cohort Analysis

**DOI:** 10.3390/jcm15103815

**Published:** 2026-05-15

**Authors:** Marco Ghisalberti, Alberto Salvicchi, Angela Galgano, Rossella Reale, Chiara Catelli, Luca Luzzi, Piero Paladini

**Affiliations:** 1 Thoracic Surgery and Lung Transplant Unit, Azienda Ospedaliero-Universitaria Senese, 53100 Siena, Italy; marco.ghisalberti@ao-siena.toscana.it (M.G.);; 2 Department of Medicine, Surgery and Neurosciences, University of Siena, 53100 Siena, Italy

**Keywords:** lung adenocarcinoma, histological pattern, second predominant component, prognosis, overall survival, disease-free survival, IASLC grading, lepidic, micropapillary

## Abstract

**Background:** Lung adenocarcinoma is morphologically heterogeneous, composed of various histological patterns. The International Association for the Study of Lung Cancer (IASLC) grading system, based on the predominant pattern and the presence of high-grade components, underscores this heterogeneity’s prognostic relevance. However, the specific impact of the non-predominant “second component” on survival outcomes in early-stage disease remains inadequately characterized. **Methods:** We conducted a retrospective, single-center study including 95 patients with pathological stage 0, I, and II (TNM 8th edition) lung adenocarcinoma who underwent complete anatomical resection (lobectomy or segmentectomy) between January 2020 and December 2021. Histopathological evaluation followed the WHO 5th edition classification, with patterns quantified in 5% increments. The second predominant component was defined as the second most represented histological pattern, irrespective of a fixed percentage threshold. Overall survival (OS) and disease-free survival (DFS) were analyzed. **Results:** A second predominant component was identified in 55 patients (57.9%). The most common second components were lepidic (30.5%), solid (18.9%), and micropapillary (10.5%). With a median follow-up of 36 months, the presence of a lepidic second component was an independent factor for improved OS (Hazard Ratio [HR] 0.70, 95% CI 0.52–0.95, *p* = 0.022) and DFS (HR 0.62, 95% CI 0.41–0.93, *p* = 0.021). Conversely, a micropapillary second component was a strong independent predictor of worse OS (HR 1.81, 95% CI 1.24–2.64, *p* = 0.002) and DFS (HR 2.03, 95% CI 1.32–3.12, *p* = 0.001). The solid second component showed an intermediate adverse effect on DFS (HR 1.45, 95% CI 1.01–2.08, *p* = 0.043). **Conclusions:** The second predominant histological pattern provides additional prognostic information beyond the IASLC grading system and may improve postoperative risk stratification in early-stage lung adenocarcinoma. A lepidic second component portends a favorable prognosis, while micropapillary and solid components denote aggressive tumor biology and higher recurrence risk. Incorporating the evaluation of second components into routine pathological reporting and clinical decision-making could enhance postoperative risk stratification and personalize adjuvant therapy strategies.

## 1. Introduction

Lung cancer persists as the leading cause of cancer-related mortality globally, with adenocarcinoma representing the most prevalent histological subtype [1]. For patients with early-stage (I–II) non-small cell lung cancer (NSCLC), anatomical surgical resection remains the cornerstone of curative-intent treatment [2]. Nevertheless, postoperative outcomes exhibit considerable heterogeneity, with a non-negligible proportion of patients experiencing disease recurrence despite complete resection [3]. This clinical reality underscores the imperative to refine prognostic tools beyond the traditional Tumor–Node–Metastasis (TNM) staging system.

The recognition of lung adenocarcinoma’s intrinsic histological heterogeneity has been a pivotal advance. The 2021 WHO Classification of Thoracic Tumors and the subsequent IASLC grading system formally acknowledge that these tumors are frequently admixtures of distinct architectural patterns, including lepidic, acinar, papillary, solid, and micropapillary [4,5]. The prognostic hierarchy of these patterns is well-established: lepidic-predominant tumors are associated with excellent outcomes, acinar and papillary with intermediate prognosis, while solid and micropapillary patterns confer a high risk of recurrence and mortality [6,7]. The IASLC grading system effectively stratifies risk by classifying tumors as grade 1 (well-differentiated), grade 2 (moderately differentiated), or grade 3 (poorly differentiated) based on the predominant pattern and the presence of ≥20% high-grade (solid/micropapillary/cribriform) components [5].

However, a nuanced histological detail often relegated to descriptive reporting is the second predominant pattern (or “second component”). It is important to distinguish between “minor components”, often defined using arbitrary thresholds (e.g., >5%), and the “second predominant pattern”, which specifically refers to the second most represented histological component regardless of its absolute percentage. Current grading primarily considers high-grade components only when they comprise ≥ 20% of the tumor. Emerging evidence suggests that even smaller quantities of aggressive patterns, or the presence of specific non-dominant patterns, may significantly influence tumor behavior [8,9]. For instance, studies indicate that the presence of any micropapillary or solid component (>5%) in stage IA disease can worsen prognosis [10]. Conversely, a secondary lepidic component has been suggested as a favorable prognostic marker [11,12].

Despite these insights, the independent prognostic weight of the second most prevalent histological component, across all pattern types and within the context of modern IASLC grading, is not systematically defined. Clarifying this could have direct clinical implications, potentially identifying a subset of early-stage patients who, despite a favorable predominant pattern or grade, harbor a hidden aggressive biological potential deserving closer surveillance or consideration of adjuvant therapy.

The aim of this study was to evaluate whether the second predominant histological pattern provides additional prognostic information in patients with resected early-stage lung adenocarcinoma.

Both disease-free survival (DFS) and overall survival (OS) were selected as endpoints, as DFS is particularly sensitive to early recurrence dynamics in early-stage disease, while OS provides a comprehensive measure of long-term clinical outcome.

## 2. Materials and Methods

### 2.1. Study Design and Patient Selection

This was a retrospective, observational cohort study conducted at the Thoracic Surgery Unit of the Azienda Ospedaliero-Universitaria Senese. This study was conducted in accordance with the Declaration of Helsinki. Due to the retrospective nature of the study and the use of de-identified clinical data, the local Ethics Committee (Comitato Etico Area Vasta Sud Est, Toscana) confirmed that formal individual approval was not required, provided that the data were analyzed anonymously and for research purposes only.

We screened the electronic surgical and pathological databases to identify all consecutive patients who underwent anatomical pulmonary resection (lobectomy or segmentectomy) for primary lung cancer between 1 January 2020 and 31 December 2021. The inclusion criteria were: (1) histopathological diagnosis of invasive non-mucinous lung adenocarcinoma or adenocarcinoma with a minor mucinous component (a minor mucinous component was defined as a non-dominant mucinous pattern representing a limited proportion of the tumor); (2) pathological stage 0, IA1, IA2, IA3, IB, IIA, or IIB according to the 8th edition of the AJCC/UICC TNM classification [13]; (3) complete (R0) surgical resection; and (4) availability of complete clinicopathological and follow-up data.

Key exclusion criteria were: (1) receipt of neoadjuvant chemotherapy or radiotherapy; (2) incomplete resection (R1 or R2); (3) final histology other than adenocarcinoma (e.g., squamous cell carcinoma, small cell carcinoma, metastases, adenosquamous carcinoma); and (4) incomplete follow-up data or follow-up duration of less than 12 months unless an event (death/recurrence) occurred earlier.

### 2.2. Surgical and Pathological Evaluation

All surgeries were performed by experienced thoracic surgeons. The surgical approach (open thoracotomy, Video-Assisted Thoracoscopic Surgery [VATS], or Robot-Assisted Thoracoscopic Surgery [RATS]) was selected based on tumor characteristics, patient physiology, and surgeon preference. Systematic ipsilateral hilar and mediastinal lymph node dissection or sampling was performed in all cases according to ESTS guidelines [14].

Resection specimens were processed according to standard protocols. Histopathological evaluation was performed by two experienced pulmonary pathologists, blinded to the clinical outcomes. Diagnosis and subtyping adhered strictly to the criteria of the 5th edition WHO Classification of Thoracic Tumors [4]. For each tumor, the percentage of each histological pattern (lepidic, acinar, papillary, micropapillary, solid, cribriform) was estimated in 5% increments. The predominant pattern was defined as the pattern with the greatest percentage. The second predominant pattern was defined as the pattern with the second-highest percentage. Tumors with a single pattern were classified as having “no second component.” In cases where two secondary patterns were present in equal proportions, the pattern with higher recognized biological aggressiveness was selected. The IASLC grade (G1, G2, G3) was assigned based on the predominant pattern and the presence of ≥20% high-grade patterns [5]. The presence of Spread Through Air Spaces (STAS) and visceral pleural invasion (VPI) was also recorded. Molecular profiling for driver mutations (EGFR, KRAS, BRAF, ALK, ROS1) and PD-L1 immunohistochemistry (using the 22C3 pharmDx assay with a 1% cut-off) was performed as per clinical standards, and data were retrieved when available.

No formal interobserver agreement analysis (e.g., kappa statistics) was performed.

### 2.3. Follow-Up and Outcome Definitions

Postoperative follow-up was standardized. Patients were clinically evaluated and underwent contrast-enhanced chest computed tomography (CT) every six months for the first two years, and annually thereafter. Brain imaging (MRI or CT) and positron emission tomography (PET-CT) were performed in case of clinical or radiological suspicion of recurrence.

The primary endpoints were Overall Survival (OS), defined as the time from surgery to death from any cause, and Disease-Free Survival (DFS), defined as the time from surgery to the first event of either locoregional recurrence, distant metastasis, or death from any cause, whichever occurred first. Recurrences were categorized as: (a) *Local*: recurrence at the surgical staple line or bronchial stump; (b) *Regional*: recurrence in ipsilateral hilar/mediastinal lymph nodes, ipsilateral pleura, or ipsilateral lung lobes; or (c) *Distant*: recurrence in the contralateral lung, contralateral lymph nodes, or extra-thoracic organs.

### 2.4. Statistical Analysis

Continuous variables are presented as mean ± standard deviation or median with interquartile range (IQR), as appropriate. Categorical variables are presented as frequencies and percentages. Group comparisons were performed using the Student’s *t*-test, Mann–Whitney U test, Chi-square test, or Fisher’s exact test.

Survival curves for OS and DFS were constructed using the Kaplan–Meier method and compared using the log-rank test. Univariable and multivariable Cox proportional hazards regression models were employed to identify independent prognostic factors. Variables with a *p*-value < 0.10 in univariable analysis and clinically relevant factors (age, sex, pathological stage, tumor size, IASLC grade, and STAS when appropriate) were included in the multivariable model. The prognostic impact of each type of second predominant pattern (lepidic, solid, micropapillary) was analyzed in separate multivariable models, adjusted for the same set of covariates, to avoid multicollinearity. Results are reported as Hazard Ratios (HRs) with 95% confidence intervals (CIs). A two-sided *p*-value < 0.05 was considered statistically significant. All analyses were performed using SPSS Statistics version 28.0 (IBM Corp., Armonk, NY, USA).

## 3. Results

### 3.1. Patient and Tumor Characteristics

From an initial pool of 128 patients, 95 met all inclusion criteria and constituted the study cohort. The baseline clinicopathological characteristics are summarized in Table 1. The mean age was 70.2 ± 8.1 years, with a male predominance (60%). The mean tumor size was 2.3 ± 1.1 cm. Minimally invasive surgery (VATS/RATS) was performed in 68.4% of cases. Pathological stages were distributed as follows: stage 0/I (74.7%) and stage II (25.3%). The predominant histological patterns were acinar (50.5%), lepidic (24.2%), and solid (14.7%). According to the IASLC system, tumors were classified as grade 1 (26.3%), grade 2 (57.9%), and grade 3 (15.8%).

### 3.2. Prevalence and Features of the Second Predominant Pattern

A second predominant histological component was identified in 55 patients (57.9%). The distribution of these second components is detailed in Table 2. The lepidic pattern was the most frequent second component (30.5% of all second components), followed by solid (18.9%) and micropapillary (10.5%). Notably, among the 10 patients with a micropapillary second component, 7 (70%) had a primary predominant pattern other than micropapillary (e.g., acinar or papillary), and their IASLC grade was G2.

### 3.3. Survival Outcomes and Univariate Analysis

The median follow-up time for the entire cohort was 36 months (IQR: 26–41). During this period, 21 patients (22.1%) experienced disease recurrence, and 18 patients (18.9%) died.

**Figure 1:**
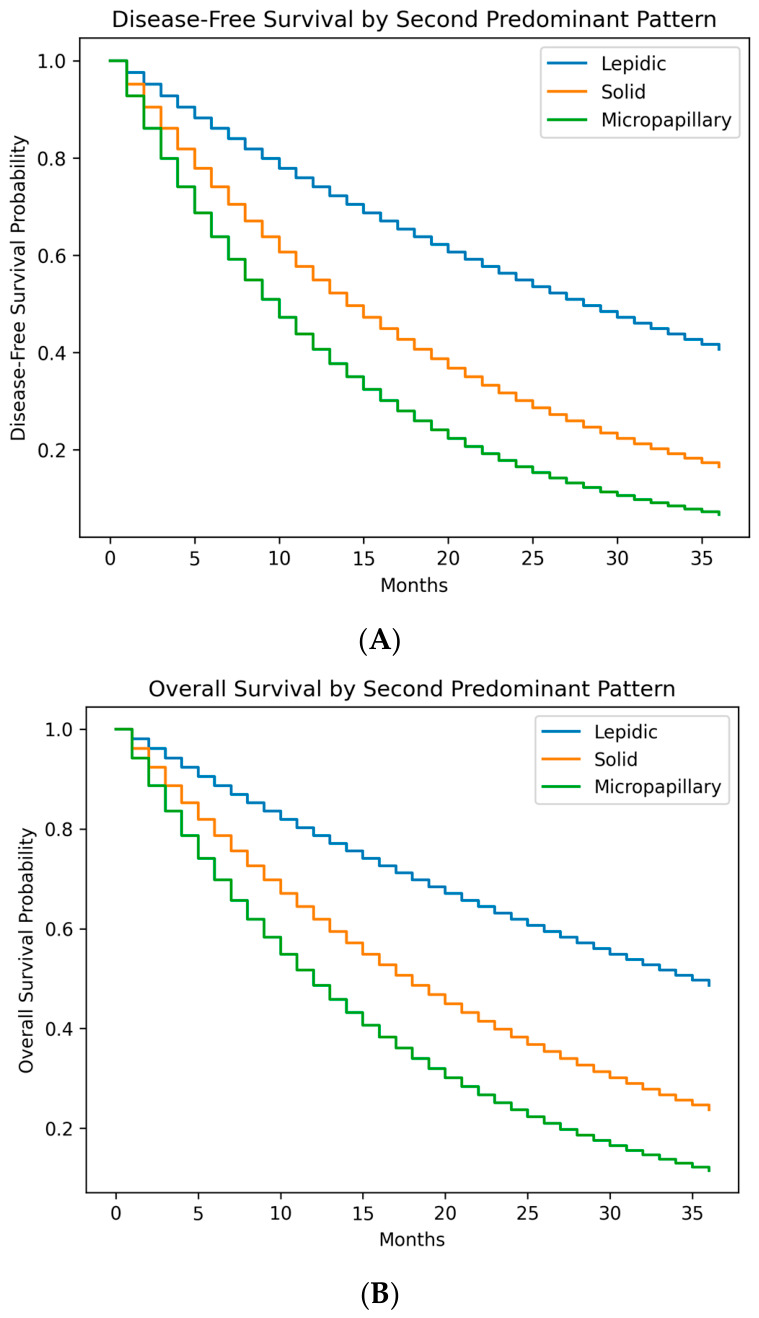
Kaplan–Meier Curves. (**A**) OS stratified by the presence of any second predominant component. (**B**) DFS stratified by the specific type of second predominant component (Lepidic vs. Solid vs. Micropapillary). The log-rank test shows a highly significant difference between groups (*p* < 0.001).

In univariate Kaplan–Meier analysis (log-rank test), the following factors were significantly associated with worse OS and/or DFS: pathological stage II (vs. 0/I), tumor size > 3 cm, IASLC grade 3 (vs. G1/G2), presence of VPI, and presence of STAS. The presence of a second predominant pattern per se was not a significant prognosticator, but the type of second component showed stark differences (Figure 1).

### 3.4. Impact of Second Predominant Pattern on Survival: Multivariable Analysis

After adjusting for age, sex, pathological stage, tumor size, and IASLC grade in Cox multivariable models, the specific type of second component emerged as an independent prognostic factor (Table 3). The mean proportion of the second predominant component was approximately 18% (range: 5–40%).

- Lepidic Second Component: Was an independent favorable prognostic factor for both OS (HR 0.70, 95% CI 0.52–0.95, *p* = 0.022) and DFS (HR 0.62, 95% CI 0.41–0.93, *p* = 0.021).
- Micropapillary Second Component: Was the strongest independent adverse prognostic factor, associated with a significantly increased risk of death (HR 1.81, 95% CI 1.24–2.64, *p* = 0.002) and recurrence (HR 2.03, 95% CI 1.32–3.12, *p* = 0.001).
- Solid Second Component: Showed a significant independent association with worse DFS (HR 1.45, 95% CI 1.01–2.08, *p* = 0.043) and a non-significant trend towards worse OS (HR 1.32, 95% CI 0.92–1.89, *p* = 0.136).

The corresponding Kaplan–Meier curves for DFS, stratified by the presence and type of second component, visually demonstrate these pronounced differences (Figure 1).

Molecular data were available for a subset of patients. Due to incomplete data availability, no formal statistical correlations were performed.

## 4. Discussion

This retrospective analysis provides compelling evidence that the second predominant histological pattern holds independent prognostic significance in patients with surgically resected early-stage lung adenocarcinoma. Our findings corroborate and extend the growing literature on tumor heterogeneity, suggesting that a binary assessment based solely on the predominant pattern or the IASLC grade (which uses a 20% high-grade threshold) may not capture the full spectrum of biological risk.

The most salient finding is the powerful negative prognostic impact of a micropapillary second component. Patients harboring this feature faced more than a two-fold increased risk of recurrence and an 80% higher risk of death, even after adjusting for stage, tumor size, and overall IASLC grade. Importantly, in our cohort, 70% of these tumors were classified as IASLC grade 2 (moderately differentiated), as the micropapillary component was present but below the 20% threshold. This aligns with recent meta-analyses and studies suggesting that any quantifiable micropapillary component (>5% or even >1%) is a biomarker of aggressive behavior, increased metastatic potential, and poor survival [10,15]. Our data strongly support the argument for reporting and actively searching for even minor micropapillary components during pathological review.

Conversely, the identification of a lepidic second component emerged as a favorable prognostic marker, associated with approximately a 30–40% reduction in the risk of death or recurrence. This “protective” effect of a secondary lepidic pattern has been observed previously [11,12] and may reflect a tumor with a more indolent growth pattern and lower propensity for invasion and metastasis.

The protective effect of a lepidic second component may reflect a tumor with lower invasive potential, preservation of alveolar architecture, and reduced propensity for vascular or lymphatic dissemination. It reinforces the concept that lepidic growth, even when non-predominant, contributes to a less aggressive tumor phenotype.

The solid second component demonstrated an intermediate adverse effect, significantly impacting DFS. This pattern is known to be associated with a high proliferative index, poor differentiation, and a propensity for distant, multisite recurrences [3]. Its presence as a secondary element likely contributes to the tumor’s metastatic fitness.

From a clinical perspective, these findings have tangible implications. First, they advocate for the mandatory and quantitative reporting of the second predominant pattern in standard pathological reports for lung adenocarcinoma. This simple addition would provide clinicians with a finer risk stratification tool. Second, they fuel the ongoing debate on adjuvant therapy in early-stage disease. Current guidelines primarily recommend adjuvant chemotherapy for stage IIB–III and selectively for stage IIA with larger tumors [16]. Our data suggest that patients with stage I or IIA disease who have a micropapillary (or, to a lesser extent, solid) second component may represent a higher-risk subgroup that could warrant closer postoperative surveillance. However, any implication for adjuvant treatment remains speculative and requires prospective validation [17]. This hypothesis warrants validation in prospective, ideally randomized, trials. The strong adverse prognostic effect of the micropapillary second component may also be biologically linked to its known association with STAS and increased metastatic potential, which could partially explain the observed outcomes.

Our findings are in line with a growing body of recent literature highlighting the prognostic relevance of tumor heterogeneity in lung adenocarcinoma. In recent years, several studies have emphasized that even limited proportions of high-grade histological patterns, particularly micropapillary and solid components, are associated with significantly worse oncological outcomes, including increased risk of recurrence and reduced survival. A recent study by Li et al. (2024) demonstrated that the presence of non-predominant micropapillary components in stage I lung adenocarcinoma is associated with poorer prognosis and may even influence the benefit derived from adjuvant treatment [18]. Similarly, contemporary analyses have confirmed that micropapillary and solid components are strongly associated with aggressive tumor behavior and higher metastatic potential [19].

Conversely, recent investigations have also reported that non-predominant lepidic components are associated with more indolent tumor behavior and improved clinical outcomes. For example, a recent multicenter study (2024) showed that tumors with non-predominant lepidic features tend to present more favorable prognostic characteristics, supporting their potential protective role [20]. This observation aligns with the hypothesis that lepidic growth reflects preserved alveolar architecture and reduced invasive capacity.

More broadly, contemporary studies have increasingly focused on refining histological risk stratification beyond the predominant pattern alone, incorporating the relative distribution of multiple architectural components within the tumor. In this context, our results further support the concept that the second predominant pattern represents a simple and reproducible parameter that may capture clinically relevant information not fully accounted for by current grading systems.

Our study has several limitations. Its retrospective, single-center design introduces potential selection and information biases. Given the limited number of events, the multivariable models may be prone to overfitting and should be interpreted cautiously. In particular, the number of events per variable was below the commonly recommended threshold for Cox regression modeling. To mitigate this, we adopted a parsimonious modeling strategy and analyzed each second predominant pattern in separate models to reduce the number of covariates. We did not perform formal model performance analyses such as Harrell’s C-index or net reclassification improvement (NRI). Given the limited sample size and number of events, these estimates would likely be unstable and potentially misleading. Therefore, our study should be interpreted as exploratory and not intended to demonstrate superiority over existing prognostic models such as the IASLC grading system.

Furthermore, no formal interobserver variability analysis was conducted, and no external central pathology review was performed. Although evaluations were carried out by experienced pulmonary pathologists according to WHO criteria, variability in pattern quantification remains a potential source of bias.

The interaction between histological patterns and molecular alterations remains an area of active investigation. For instance, EGFR-mutated tumors are often associated with lepidic features, which may partially explain the more favorable prognosis observed in this subgroup.

Although STAS was recorded and associated with outcomes in univariable analysis, it was not included in multivariable models to avoid overfitting given the limited number of events.

A central pathological review, while based on WHO criteria, was not performed in this pragmatic study. Finally, the follow-up duration, though with a median of 3 years, is relatively short for indolent adenocarcinomas; longer-term data are needed.

## 5. Conclusions

In conclusion, this study demonstrates that the second predominant histological pattern is not merely a descriptive detail but an independent prognostic determinant in early-stage lung adenocarcinoma. A micropapillary second component is a robust marker of aggressive disease and poor outcomes, while a lepidic second component signifies a more favorable prognosis. From a clinical perspective, the evaluation of the second predominant pattern may represent a simple and reproducible tool to refine postoperative risk stratification. However, these findings should be considered hypothesis-generating and require validation in larger, prospective cohorts before influencing therapeutic decision-making. Future research should focus on validating these findings in larger, multi-institutional cohorts and exploring the potential of this histological feature to guide personalized adjuvant treatment strategies in early-stage lung cancer.

## Figures and Tables

**Table 1:** Baseline clinicopathological characteristics of the study cohort (n = 95).

Characteristic	Value
Demographics	
Age, years (mean ± SD)	70.2 ± 8.1
Male Sex, n (%)	57 (60.0)
Surgical Data	
Surgical Approach, n (%):	
VATS/RATS	65 (68.4)
Open Thoracotomy	30 (31.6)
Type of Resection, n (%):	
Lobectomy	82 (86.3)
Segmentectomy	13 (13.7)
Pathological Data	
Tumor Size, cm (mean ± SD)	2.3 ± 1.1
Pathological Stage (8th ed.), n (%):	
0/IA1–IA3	56 (58.9)
IB	15 (15.8)
IIA/IIB	24 (25.3)
Predominant Pattern, n (%):	
Acinar	48 (50.5)
Lepidic	23 (24.2)
Solid	14 (14.7)
Papillary	7 (7.4)
Micropapillary	3 (3.2)
IASLC Grade, n (%):	
G1	25 (26.3)
G2	55 (57.9)
G3	15 (15.8)
Visceral Pleural Invasion, n (%)	18 (18.9)
STAS present, n (%)	22 (23.2)

**Table 2:** Distribution of Second Predominant Histological Patterns.

Second Predominant Pattern	n	% of Total Cohort	% of Patients with a 2nd Component
Lepidic	29	30.5	52.7
Solid	18	18.9	32.7
Micropapillary	10	10.5	18.2
Acinar	8	8.4	14.5
Papillary	0	0	0
Any Second Component	55	57.9	100
No Second Component	40	42.1	-

**Table 3:** Multivariable cox regression analysis for overall and disease-free survival (separate models for each second component type, adjusted for age, sex, pStage, tumor size > 3 cm, and IASLC Grade).

Model/Variable	Overall Survival		Disease-Free Survival	
	HR (95% CI)	*p*-Value	HR (95% CI)	*p*-Value
Model 1: Lepidic 2nd Comp.				
Lepidic Second Component	0.70 (0.52–0.95)	0.022	0.62 (0.41–0.93)	0.021
Pathological Stage II (vs. 0/I)	2.11 (1.30–3.42)	0.002	2.45 (1.55–3.88)	<0.001
IASLC Grade 3 (vs. G1/G2)	1.85 (1.15–2.98)	0.011	2.10 (1.38–3.20)	0.001
Model 2: Solid 2nd Comp.				
Solid Second Component	1.32 (0.92–1.89)	0.136	1.45 (1.01–2.08)	0.043
Pathological Stage II (vs. 0/I)	2.25 (1.42–3.56)	0.001	2.58 (1.70–3.91)	<0.001
IASLC Grade 3 (vs. G1/G2)	1.78 (1.08–2.93)	0.024	2.05 (1.31–3.21)	0.002
Model 3: Micropap. 2nd Comp.				
Micropap. Second Component	1.81 (1.24–2.64)	0.002	2.03 (1.32–3.12)	0.001
Pathological Stage II (vs. 0/I)	2.02 (1.27–3.22)	0.003	2.33 (1.55–3.51)	<0.001
IASLC Grade 3 (vs. G1/G2)	1.65 (0.99–2.75)	0.055	1.82 (1.15–2.89)	0.011

## Data Availability

The datasets generated and analyzed during the current study are not publicly available due to patient privacy regulations but are available from the corresponding author upon reasonable request.
